# Effect analysis of medial bracing plate combined with cannulated screw in unstable femoral neck fracture assisted by surgical hip dislocation: a retrospective study

**DOI:** 10.1186/s13018-023-03991-3

**Published:** 2023-07-14

**Authors:** Xin Sun, Gang Yi, Liang Ao, Xin Zhou, Tao Zhang, Tai-yuan Guan

**Affiliations:** 1 Department of Orthopedics, The Affiliated Traditional Chinese Medicine Hospital of Southwest Medical University, Luzhou, Sichuan China; 2 College Of Integration Of Traditional Chinese And Western Medicine To Southwest Medical University, Luzhou, Sichuan China

**Keywords:** Surgical hip dislocation, Femoral neck fracture in young adult, Inner support steel plate

## Abstract

**Background:**

Unstable femoral neck fractures have a high likelihood of causing severe disruption to the blood supply. This study aimed to assess the therapeutic effect of surgical hip dislocation using a medial support plate combined with cannulated screw fixation for the treatment of unstable femoral neck fractures in young and middle-aged adults.

**Methods:**

We retrospectively analyzed the medical records of 68 young adults who underwent internal fixation of unstable femoral neck fractures. The observation group included 32 patients who had received medial support plate and cannulated screw fixation by the surgical hip dislocation method and 36 patients who had undergone anti-rotation screw composite compression system fixation comprised the comparison group. The amount of intraoperative bleeding, surgery duration, fracture recovery time and complications were recorded. The degree of femoral neck shortening and Garden index were assessed using the Zlowodzki method. Additionally, hip functionality was evaluated using the Harris score at 3 and 6 months and at the last follow-up.

**Results:**

All 68 patients in both groups were followed up for 12–42 months (mean, 22.4 months). The postoperative incision was well-aligned and no inflation was observed. The intraoperative blood loss and surgery duration in the comparison group were longer than those in the observation. Additionally, the observation group had a significantly shorter fracture recovery time and a higher Garden index than the comparison at 6 months postoperatively; however, there was no significant statistical discrepancy between the two groups at the remaining time points. The observation group had higher Harris scores than the comparison at 3 and 6 months postoperatively.

**Conclusion:**

Surgical hip dislocation applied to the medial support plate combined with cannulated screw fixation has clinical application value in restoring the stability of femoral neck fractures while facilitating the maintenance of blood flow to the femoral head and neck.

## Background

Femoral neck fractures (FNF) are intra-articular capsule fractures [1, 2]. Approximately 57% of the patients with hip fractures and 3% of young and middle-aged patients have FNF. In recent years, FNF occurrence in youth has increased due to high-energy trauma, which leads to higher shear forces in the vertical direction of the femoral neck, resulting in unstable fractures [1, 3]. This has become one of the clinical difficulties in treatment.

Additionally, owing to the anatomic specificity of trophic vessels in the femoral head and neck, the likelihood of fracture malunion and avascular necrosis of the femoral head (ANFH) is higher in the later stages in FNF than in other fractures [4, 5]. Although, the principle of "preservation of the own hip joint" in treating FNF in young and middle-aged adults has been recognized by most scholars, many controversies remain regarding the surgical strategy, implant selection, and fixation structure [6–8]. Therefore, there is an ongoing exploration of anatomical repositioning and effective internal fixation with minimal destruction of the blood supply to the head of the femur.

Anti-rotation and compression internal fixation methods are widely used in hip preservation surgery; however, the postoperative femoral head necrosis rate remains high, ranging from 10%–43%, and the fracture non-healing rate ranges from 8.9 to 40% [9, 10]. In 2015, Mir and Collinge [11] proposed the use of a medial bracing plate (MBP) to enhance resistance to vertical shear of the fracture for stability, which has been increasingly validated by biomechanical experiments and applied in clinical practice [12]. Based on research on the blood supply of the femoral head, Ganz et al. designed a new surgical approach for hip joint lesions—the surgical hip dislocation (SHD) procedure—which has been confirmed to be safe and reliable [13, 14, 33]. This procedure provides a new perspective. In this study, the SHD technique was used to treat middle-aged and young adults with unstable FNF using an MBP in combination with traditional cannulated screw. A comparison of the efficacy of an MBP combined with cannulated screw fixation and anti-rotation screw composite compression system fixation in the treatment of unstable FNF in youths has rarely been reported.

We aimed to observe the efficacy of the SHD procedure applied to a medial support plate combined with cannulated screw fixation and compare it with the fixation of the anti-spin screw composite compression system.

## Methods

### Patient selection criteria

The inclusion criteria were as follows: (1) age 18–55 years; (2) diagnosis of unstable FNF confirmed by preoperative radiography and computed tomography (CT); (3) closed, unilateral FNF; (4) informed about the therapy program and willing to sign a consent form for surgical procedures; and (5) access to scheduled complete follow-up visits.

The exclusion criteria were as follows: (1) other systemic fractures; (2) previous history of hip disease, deformity, or surgery; and (3) old pathological fractures in the femur.

Between January 2018 and January 2021, 68 patients who met the inclusion criteria were selected. Among them, a medial support plate combined with cannulated screw fixation via an SHD procedure was used in 32 cases (observation group) and an anti-rotation screw combined with compression system fixation was used in 36 cases (comparison group).

### General information

In the two groups, preoperative radiography and CT screening were conducted, and Pauwels' classification was type III [15]. The observation group consisted of 19 males and 13 females aged 20–52 years, with an average age of 36.62 years. The causes of injuries were as follows: 6 cases of high-drop injuries, 11 cases of road traffic injuries, and 15 cases of fall injuries. The duration of injury to surgery was 1–5 days, with a mean of 2.7 days. There were 21 left hip fractures, 11 right hip fractures, 22 type III fractures, and 10 type IV fractures, according to the Garden classification. The comparison group consisted of 21 males and 15 females aged 22–54 years, with an average age of 35.86 years. The causes of injury were as follows: 5 cases of high-drop injuries, 13 cases of road traffic injuries, and 18 cases of falls.

### Surgical methods

After admission, the patient underwent routine preoperative examination, which included traction and braking of the affected limb with a leather sleeve, prevention of venous thrombosis of the lower limb, and pulmonary function exercise. Second-generation cephalosporins were applied preventatively 30 min before the procedure. General anesthesia or combined spinal-epidural anesthesia was administered, and the operating area was routinely disinfected and toweled.

#### Anti-rotation screw compound pressurization system group (comparison group)

**Fig. 1:**
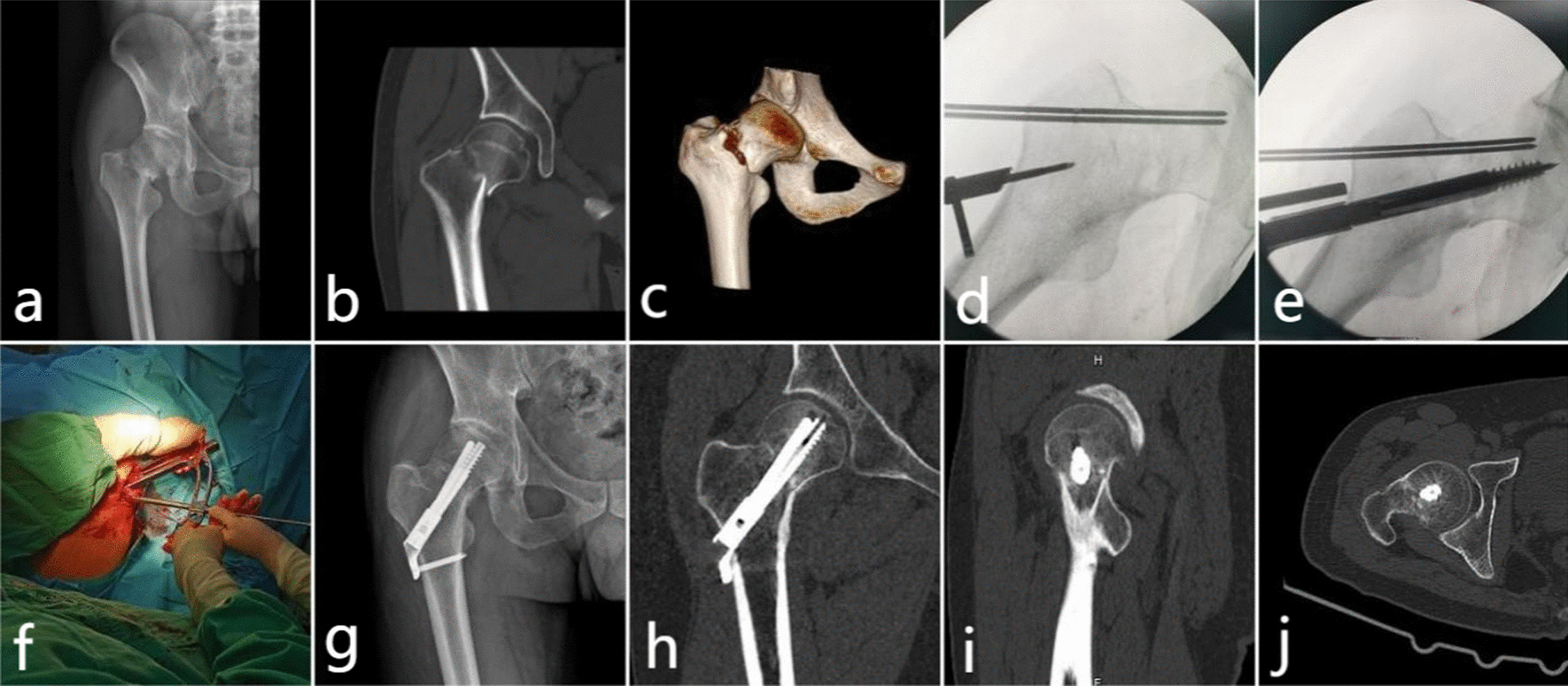
Comparison group, male, 42 years old, right femoral neck fracture caused by high falling injury (Garden III, Pauwels III) **a** preoperative positive radiograph; **b** Preoperative coronal computed tomography (CT) scan; **c** Preoperative CT plain scan in anomalous position; **d** During the operation, the guide pin locator was placed at the screw entry point, and the initial guide pin was placed; **e, f** intraoperative insertion of lag screws and anti-rotation screws; **g** Postoperative positive radiograph; **h** Postoperative coronal CT plain scan; **i** Postoperative plain sagittal CT scan; **j** Plain CT scan was performed after operation

The patient was placed supine on the traction bed with the affected limb slightly elevated, and a longitudinal incision of approximately 8 cm was made through the anterior hip approach, after which the broad fascial tensor and suture muscles were bluntly separated, and avoided trauma to the lateral femoral cutaneous nerve. After the joint capsule was dissected to fully expose the fracture, it was repositioned anatomically under direct visualization and temporarily fixed using 2 kerf pins. The pin was positioned in the central axis of the femoral neck, and the pin aligner was used to adjust the pin to a satisfactory position. Next, the depth finder was slid along the guide pin, the depth on the depth finder was read, and the appropriate lengths for the tension screw and anti-rotation screw were selected. After the limit knob was tightened, the assembled aiming frame and tension screw were slowly screwed into the femoral neck along the guide pin, and the two temporarily fixed kerf pins were removed. The main nail placement bar was loosened and removed, the anti-rotation screw bit was inserted into the aiming frame, and the hole was drilled up to the limit. The anti-rotation screw driver was used to attach the anti-rotation screw and screen it through the aiming frame. Finally, a small incision was made at the locus, and the skin and bone surfaces were bluntly separated. A plasma drain was placed, and the incision was flushed and closed layer-by-layer (Fig. 1).

#### Medial support plate combined with cannulated screw fixation via an SHD procedure (observation group)

**Fig. 2:**
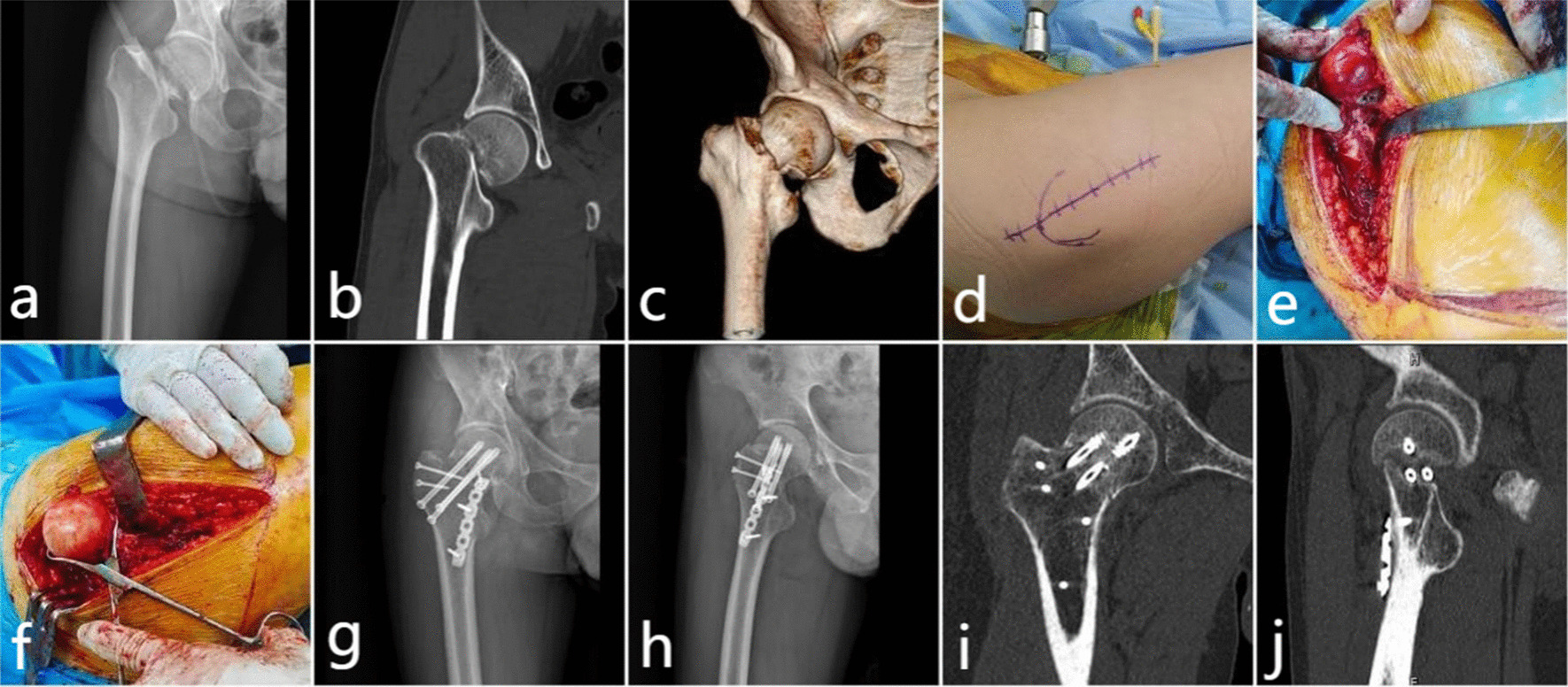
Observation group, male, 38 years old, had left femoral neck fracture caused by falling (Garden III, Pauwels III) **a** preoperative anteroposterior radiograph showed femoral neck fracture with displacement; **b, c** preoperative computed tomography (CT) plain scan and reconstruction showed the shape and instability of femoral neck fracture; **d** Access surface marking; **e, f** femoral head and neck exposed after surgical dislocation of hip joint; **g, h** six months after operation, the radiogragh of the anterior and lateral position showed that the fracture healed well and the internal fixation was in place; **i, j** six months after operation, CT plain scan of the coronal and abnormal position showed that the fracture healed well and the internal fixation was in place

The patient was placed on the healthy side after anesthesia. The first step was to perform a SHD procedure, exposing the trochanter and osteotomizing the rotor with a swing saw to a thickness of approximately 1.5 cm. The gluteus medius—greater trochanteric flap—lateral femoral muscle was pulled forward as a whole, and the iliac capsule muscle and gluteus minimus muscle were dissected to reveal the joint capsule. The joint capsule was incised in a "Z" shape to remove intra-articular bruising and hematoma. At this point, the crucial step is to enter three Kirschner wires from the outside of the femur and pass through the femoral neck to preliminarily fix the fractured end, restoring initially stability to the femoral neck, and hip dislocation was performed to reveal the femoral head and neck. In the second step, the FNF was repositioned and kerf pins were placed. The femoral neck fracture was repositioned under 360° direct vision, and three 2.0 mm kerf pins were drilled through the femoral neck to the subtrochanteric region under direct vision in the inferior lateral cortex of the femoral trochanter. The position and distribution of the kerf pins were confirmed fluoroscopically to serve as guide pins for the cannulated screw placement. The third step involved fixing the femoral neck MBP using a 1/3 tubular or pelvic reconstruction plate, which was pre-bent and placed into the anterior medial femoral neck to confirm the effectiveness of the fixation. In the fourth step, after the femoral neck MBP was well placed, the hip joint was repositioned, and 6.5 half-threaded cannulated screws were screwed into the pre-fixed kerf pins. The femoral neck MBP and cannulated screws were confirmed to be in a good position under fluoroscopy, and the greater trochanteric osteotomy block was repositioned and cross-fixed with 2–3 fully threaded cortical screws. Fracture repositioning and internal fixation were confirmed through fluoroscopy (Fig. 2).

### Postoperative management

Second-generation cephalosporins was used 24 h postoperatively to prevent infection, and calcium natriuretic heparin was administered to prevent deep vein thrombosis in the lower extremities. The plasma drainage tube was expulsed after 24–48 h, depending on the drainage flow, and the affected extremity was maintained in a neutral position with a postoperative thong shoe and maintained in traction for 6–8 weeks with about 5 kg. The patient was instructed to perform ankle pump and quadriceps isometric training on the first day after surgery, remove traction and perform hip and knee flexion and extension activities, with regular follow-up. At 12 weeks postoperatively, the patient was advised to bear weight partially on the ground using a double crutch, depending on the progress of the fracture's healing, and gradually transitioned to full weight-bearing.

### Efficacy evaluation index

In both groups, intraoperative bleeding, operative time, fracture healing time, and complications such as fracture nonunion, femoral head necrosis, nail retraction, internal fixation cutting, and fracture occurrence were recorded. Harris scores [16] (including deformity, functional activities such as sitting in a chair, tying shoelaces, walking upstairs, gait, auxiliary function during walking, and pain) were assessed for hip function at 3 and 6 months and at the final follow-up in both groups after surgery. The Zlowodzki method [17] was used to assess the degree of femoral neck shortening, and the Garden index [18] was used to assess fracture reduction. The normal orthopantomogram of the hip shows a 160° angle between the medial femoral trabeculae and the internal cortex of the femoral neck, while the lateral femoral head and the femoral neck at 180°. A Garden's index of less than 155° or more than 180°in the postoperative orthotropic or lateral position indicated poor repositioning.

### Statistical analysis

The analysis was conducted using SPSS version 20.0 software (SPSS Inc, Chicago, IL, USA). The count data is represented as ''n'' and the measurement data as mean ± standard deviation. A *t*-test was used for comparing measurement data across groups, and the chi-square test was used for comparing count data across groups. The significance level for the tests was set at α = 0.05.

## Results

There were no statistically significant differences between the two groups in terms of age, sex, cause of injury, time from injury to surgery, side of hip injury, or preoperative Garden and Pauwels’ fracture types (*P* > 0.05; Tables 1 and 2). All 68 patients in both groups were followed up for 12–42 months (mean, 22.4 months). The incision healed after surgery at stage I (wounds with neat edges, no infection, and tightly closed trabeculae). In the observation group, the operative duration varied from 75 to 125 min (average, 94 min), and the intraoperative bleeding volume ranged from 105 to 240 mL (mean, 155 mL). The comparison group had a mean operation time of 88 min (range, 60–105 min) and a mean intraoperative bleeding volume of 126 mL (range, 85–150 mL). The amount of intraoperative bleeding and operative time were significantly longer in the comparison group than in the observation group (*P* < 0.05). The observation group had a shorter recovery time for fractures than the comparison group. The differences between the two groups were statistically significant (*P* < 0.05). According to the Garden Index classification, the observation group had anatomical and acceptable repositioning after surgery; the observation group showed only one instance of poor repositioning following surgery, and their Garden Index was considerably better than that of the comparison group six months after surgery. However, there were no statistically significant differences between the two groups at the other time points (Tables 3). The Harris scores for both groups showed a significant improvement at all postoperative periods compared to before the surgery (*P* < 0.05), and the disparity in time intervals also had statistical significance (*P* < 0.05). Additionally, the observation group showed significantly higher Harris scores compared to the comparison group at 3- and 6-month post-surgery marks (*P* < 0.05; Tables 4). During the follow-up period, the observation group encountered two instances of femoral neck shortening, no occurrences of unhealed fractures, and one case of femoral head necrosis. Conversely, the comparison group experienced six cases of femoral neck shortening, three cases of unhealed fractures, and three cases of femoral head necrosis. Joint replacements for femoral neck shortening, fracture nonunion, and femoral head necrosis were the solutions for six patients in the comparison group (Tables 5).

**Table 1:** Comparison of general data between the two groups (*x* ± s)

Group	Gender (cases)		Age (years)	Affected side (cases)		Garden typing		Causes of injury (cases)			Time from injury to
	Male	Female		Left	Right	III	IV	High fall	Traffic	Fall	
Observation group	19	13	36.62 ± 10.55	21	11	22	10	6	11	15	2.74 ± 0.62
Comparison group	21	15	35.86 ± 9.38	21	15	20	16	5	13	18	2.63 ± 0.58
Statistic	*χ*^2^ = 1.069		*t* = 0.815	*χ*^2^ = 1.171		*χ*^2^ = 1.456			*χ*^2^ = 0.661		*t* = 1.146
	*P* = 0.301		*P* = 0.418	*P* = 0.279		*P* = 0.654			*P* = 0.801		*P* = 0.255

**Table 2:** Comparison of clinical indicators between the two groups

Group	*n*	Operation time (min)	Intraoperative bleeding volume (ML)	Fracture healing time (week)
Observation group	32	94.76 ± 10.52	155.88 ± 15.64	14.47 ± 2.18
Comparison group	36	88.30 ± 6.48	126.45 ± 12.34	18.60 ± 4.93
Statistic		*t* = 5.940	*χ*^2^ = 0.248	*t* = 5.044
		*P* = 0.092	*P* = 0.619	*P* = 0.000

**Table 3:** Comparison of Garden score between the two groups

Group	*n*	Positive position			Lateral position		
		3 months after	6 months	Latest follow	3 months	6 months after	Latest follow
Observation group	32	79.5 ± 6.4^#^	77.2 ± 8.8*	87.8 ± 8.4^#^*	87.7 ± 4.2^#^	83.5 ± 6.9*	90.8 ± 7.6^#^*
Comparison group	36	76.3 ± 4.9^#^	73.4 ± 5.3*	83.5 ± 7.6^#^*	86.5 ± 6.4^#^	82.1 ± 7.3*	90.3 ± 9.0^#^*
Statistic		*t* = 2.234	*t* = 2.065	*t* = 2.149	*t* = 0.905	*t* = 0.794	*t* = 0.243
		*P* = 0.029	*P* = 0.043	*P* = 0.035	*P* = 0.369	*P* = 0.430	*P* = 0.809

*Compared with the value at 3 months after the operation, *P* < 0.05; ^#^compared with the value at 6 months after the operation, *P* < 0.05

**Table 4:** Comparison of Harris score of the hip joint before and after operation between the two groups

Group	*n*	Preoperative	3 months after operation	6 months after operation	Latest follow-up	Statistic
Observation group	32	1.95 ± 0.30^#αβ^	3.95 ± 0.48*^αβ^	5.16 ± 0.61*^#β^	8.14 ± 0.77*^#αβ^	*F* = 805.128 *P* = 0.000
Comparison group	36	1.88 ± 0.72^#αβ^	3.18 ± 0.45*^αβ^	4.98 ± 0.70*^#β^	7.93 ± 0.80*^#αβ^	*F* = 526.771 *P* = 0.000
Statistic		*t* = 0.647	*t* = 7.684	*t* = 1.292	*t* = 1.240	
		*P* = 0.521	*P* = 0.000	*P* = 0.200	*P* = 0.219	

*Compared with preoperative value, *P* < 0.05; ^#^compared with the value at 3 months after the operation, *P* < 0.05; α: compared with the value at 6 months after the operation, *P* < 0.05; β: compared with the value at 9 months after the operation, *P* < 0.05

**Table 5:** Comparison of complications between the two groups

Group	*n*	Femoral neck shortening (cases)	Nonunion of fracture (cases)	Avascular necrosis of femoral head (cases)
Observation group	32	2	0	1
Comparison group	26	6	3	3
Statistic		*χ*^2^ = 0.248	*χ*^2^ = 0.578	*χ*^2^ = 0.954
		*P* = 0.000	*P* = 0.000	*P* = 0.000

## Discussion

According to this study's findings, the Harris scores significantly improved in both groups at all postoperative time points compared with the preoperative scores, intraoperative bleeding and operative time were better in the comparison group than in the other group. However, the observation group exhibited a shorter fracture healing time and had a higher Garden index at 6 months after surgery and higher Harris scores at 3 and 6 months after surgery. Although MBP combined with cannulated screw fixation for surgical dislocation of the hip joint did not have advantages in terms of surgical bleeding and operative time, the outcomes after surgery were superior in maintaining the anatomical position of the fracture, supporting functional recovery, and reducing the healing time. It can be identified that compared with the treatment of internal fixation using anti-rotation compression screws alone, the addition of surgical dislocation of the hip joint and an MBP increases intraoperative bleeding and operative time, cannulated screw fixation is prone to femoral neck shortening, deformed healing, and fixation failure.

High-energy-induced unstable FNF is prone to serious complications such as ANFH and fracture nonunion due to severe blood supply disruption and significantly displaced fractures. As with fractures in general, anatomical repositioning and strong interior fixation are imperative for fracture healing. Currently, three cannulated screws fixation remains the preferred method of managing general FNF by domestic and international scholars [19, 20]. However, in unstable FNF, cannulated screw fixation is prone to femoral neck shortening, deformed healing, and fixation failure.

Previous studies suggest that the successful use of internal fixation to enhance the shear resistance of FNF is the key to the success of this procedure [11, 12, 21–24]. The composite compression system of anti-rotation screws consists of a tension nail, anti-rotation nail, fixation plate, and sliding compression nail, which have good angular and rotational stability. It can resist certain torsional and twisting forces and provides favorable conditions for fracture healing. Since 2015, MBP has been validated by an increasing number of biomechanical experiments in resisting vertical shear of fractures. Additionally, MBP has been found to have the advantages of good functional recovery and short fracture recovery time, and few postoperative complementary diseases have been found through clinical application to promote fiber healing [25].

In addition to anatomical repositioning and strong internal fixation, the protection of blood supply to the femoral head poses a great challenge for FNF with special anatomical locations [26, 27]. The results indicated that the medial femoral circumflex artery (MFCA) was the main source of blood supply. One study demonstrated by MFCA alignment that approximately 32.2% originated in the femoral artery and about 64.6% in the deep femoral artery [28]. Most FNF fractures in young and middle-aged adults are caused by high-energy violent injuries, resulting in displaced fractures. For patients with vertical instability of FNF fractures, obtaining a satisfactory and stable closed reduction of the displaced FNF fracture using manipulation or mechanical methods is difficult [29]. Therefore, sectional reduction has become the first choice and is emphasized upon in the recommendations for managing these fractures [30].

The traditional surgical approaches for hip fracture reduction mainly include the Smith-Peterson (S-P) anterolateral, Kocher–Langenbeck (K-L) posterolateral, and Watson-Jones (W-J) anterolateral approaches. The W-J approach and the modified S-P approach are still the dominant surgical access options for FNF in youth reported to date. However, owing to the special disposition of the blood supply to femoral neck, the use of these two intraoperative approaches aggravates vascular damage leading to complications such as holding dysfunction and sequential pain, collapse of the femoral head, and ANFH [31, 32]. Ganz et al. [33] proposed an innovative approach to SHD using a hip dislocation technique combined with osteotomy reversal of the femoral trochanter.

The use of surgical dislocation techniques in the hip joint has been reported [13, 14], and has shown that the current "hip preservation technique" surgical approach is the safest and most effective in surgical dislocation of the hip joint. This technique minimizes damage to the MFCA and reduces the possibility of postoperative ANFH while ensuring adequate exposure of the operative area. Some studies [6, 12] have reported good clinical results using MBP combined with cannulated screw technique in the treatment of middle-aged and young adults with unstable FNF. Their results also proved that this technique can provide excellent fixation strength, ensure good repositioning, reduce complications, and promote functional rehabilitation of the affected limb after surgery. Therefore, it is an excellent choice for the treatment of FNF in young and middle-aged adults.

The combination of MBP and cannulated screw fixation for surgical dislocation of the hip is more aligned with the biological principle as it adds an MBP while retaining the anti-rotation ability of the hollow helix. This method can effectively disperse the shear stress at the fracture end, enhance the fixation effect, strongly maintain the dissection position of the fragment, and promote patient recovery. The groups demonstrated substantial progress in their hip function scores and Garden index postoperatively, and their effectiveness within a medium- to long-term timeframe was considered satisfactory, with the observation group showing better clinical results.

The reason for this variation is the unique anatomical and physiological structure of the femoral neck, which is subjected to great shear stress and rotational force under weight-bearing conditions. The internal fixation of the composite compression system with anti-rotation screws can address rotational displacement of the fracture end after FNF and compress the fracture end axially; however, it lacks anti-shear stresses. A better solution is to supplement screw fixation with an MBP. The traditional cannulated screw provides compression and rotation resistance to the fracture end, and the support plate counteracts the shear stress from the weight bearing and converts the shear force into compression at the fracture end, providing maximum fixation strength and stability [32, 34]. The subsequent examinations demonstrated that the likelihood of complications was lower in the observation group than in the comparison group. This confirms that the use of an MBP, along with the cannulated screw fixation technique during the surgical dislocation of the hip, did not impact the femoral head's blood supply or the FNF dissection. Moreover, it provided more favorable conditions for fracture healing, and its advantages far outweighed its shortcomings.

This study had certain limitations. First, this study was not a randomized controlled trial (RCT), and the follow-up duration was relatively short. More RCTs and prospective studies are warranted to further assess and compare distant efficacy, especially distant femoral head necrosis. Second, the SHD approach requires a certain technical learning curve, and there are still few reported cases in this modality at home and abroad. Thus, more cases are warranted to validate the clinical benefits. Third, the main postoperative complication of SHD in this study was ANFH; however, the cause of ANFH was related to the type of fracture and time of injury, and no clarification could be determined with this surgical approach. No further research has been conducted on the statistical effect of data on femoral neck fracture rates on boiling and avascular necrosis rate.

## Conclusion

Compared with the treatment of unstable FNF in young and middle-aged adults with anti-rotation screws and a compression system, SHD applied to a medial support plate combined with cannulated screw fixation provides better surgical exposure and avoids damage to the blood supply to the femoral neck while restoring the stability of FNF. This significantly reduces the incidence of fracture re-displacement, fracture nonunion, and ischemic necrosis of the femoral head and has a high clinical application value.

## Data Availability

The datasets analyzed during the current study are available from the corresponding author on reasonable request.

## Abbreviations

FNF: Femoral neck fracture
ANFH: Avascular necrosis of the femoral head
MBP: Medial bracing plate
SHD: Surgical hip dislocation
MFCA: Medial femoral circumflex artery
RCT: Randomized controlled trial
CT: Computed tomography
